# Re-Epithelialization Appraisal of Skin Wound in a Porcine Model Using a Salmon-Gelatin Based Biomaterial as Wound Dressing

**DOI:** 10.3390/pharmaceutics11050196

**Published:** 2019-04-26

**Authors:** Cristian A. Acevedo, Elizabeth Sánchez, Nicole Orellana, Patricio Morales, Yusser Olguín, Donald I. Brown, Javier Enrione

**Affiliations:** 1 Centro de Biotecnología, Universidad Técnica Federico Santa María, Avenida España 1680, Valparaíso 2340000, Chile; cristian.acevedo@usm.cl (C.A.A.); elizabeth.sanchez@usm.cl (E.S.); nicole.orellana@usm.cl (N.O.); patriciovet@hotmail.com (P.M.); yusser77@gmail.com (Y.O.); 2 Departamento de Física, Universidad Técnica Federico Santa María, Avenida España 1680, Valparaíso 2340000, Chile; 3 Instituto de Biología, Universidad de Valparaíso, Avenida Gran Bretaña 1111, Valparaíso 2340000, Chile; donald.brown@uv.cl; 4 Biopolymer Research and Engineering Lab, Facultad de Medicina, Universidad de los Andes, Monseñor Álvaro del Portillo 12455, Las Condes, Santiago 7550000, Chile; jenrione@uandes.cl

**Keywords:** wound healing, porcine model, salmon-gelatin biomaterial, in vivo trial

## Abstract

The design of new functional materials for skin tissue engineering is an area of constant research. In this work, a novel wound-dressing biomaterial with a porous structure, previously formulated using salmon-gelatin as main component (called salmon-gelatin biomaterial (SGB)), was tested in vivo using pigs as skin wound models. Four weeks after cutaneous excision and implantation in the animals, the healing process did not show apparent symptoms of inflammation or infection. Interestingly, the temporal evolution of wound size from 100% to around 10% would indicate a faster recovery when SGB was compared against a commercial control. Histological analysis established that wounds treated with SGB presented similar healing and epithelialization profiles with respect to the commercial control. Moreover, vascularized granulation tissue and epithelialization stages were clearly identified, indicating a proliferation phase. These results showed that SGB formulation allows cell viability to be maintained. The latter foresees the development of therapeutic alternatives for skin repair based on SGB fabricated using low cost production protocols.

## 1. Introduction

In skin tissue engineering, the pursuing of efficient, sustainable and safe alternative biomaterials is an area of constant research [1,2]. One of the strategies that has shown promising results in skin repair is the manufacturing of porous, biocompatible and biodegradable scaffolds, which imitate the extracellular matrix of the target tissue in which cells and growth factors can be incorporated allowing native proliferation and restitution of damaged tissue [3].

Various materials have been used for the fabrication of scaffolds aimed at tissue engineering applications [4,5]. Gelatin, as a denatured macromolecule from collagen, contains native valuable aminoacid sequencing. It is also a bioactive biopolymer that offers well known features for an effective constitution of functional materials such as nanoparticles, microparticles, 3D scaffolds, electrospun nanofibers and in situ gelling formulations [6]. Broadly, two types of gelatin have been studied for the conformation of biomaterials for tissue regeneration, originating from mammals and from fish [6,7]. In this regard, due of the risk of transmission to humans of certain diseases such as bovine spongiform encephalopathy and foot-and-mouth disease (both from mammalian sources), the gelatin extracted from fish offers an alternative in terms of biosecurity, which has led to a growing interest in research and development of materials formulated from this source [7,8,9]. Moreover, gelatin from cold-adapted species such as salmon (*Salmon salar*) offers the advantage for composite fabrication due to its low viscosity at ambient temperature due to is smaller average molecular weight and lower gelling temperature compared to those from mammals.

We have previously published the development of a novel wound-dressing formulated biomaterial based on salmon-gelatin [10]. This work included physical characterization and in vivo testing using a rabbit model. These experiments proved the potential use of this biomaterial in terms biosafety, however the use of a rabbit model limits the conclusions regarding its effectiveness of the scaffold. Due to the anatomical conformation, thickness and healing mechanism, an in vivo model based on porcine skin would allow a more adequate, effective and more definitive preclinical assessment tool for the development of a novel biomaterial for skin tissue engineering [11,12,13].

In this work, a novel biomaterial based on salmon-gelatin (called here salmon-gelatin biomaterial (SGB)) was formulated as a wound-dressing and tested in vivo using pigs as skin wound models. Our results include histological measurements and determination of scarring formation kinetics after four weeks of treatment.

## 2. Materials and Methods

### 2.1. Materials

Gelatin was extracted from salmon skins according to the methodology described in Enrione et al. [10]. Chitosan (pharmaceutical grade, 95% deacetylated, 300 kDa, derived from crab shells) was purchased from Quitoquimica (Concepción, Chile). Agarose (molecular biology grade) was purchased from Lonza (Morristown, NJ, USA). Glycerol (pharmaceutical grade) was purchased from Merck (Darmstadt, Germany). EDC (*N*-ethyl-*N*’-(3-dimethylaminopropyl)carbodiimide hydrochloride), NHS (*N*-hydroxysuccinimide) and MES (2-(*N*-morpholino)ethanesulfonic acid hydrate) were purchased from Sigma-Aldrich (St. Louis, MO, USA).

### 2.2. Preparation of Salmon-Gelatin Biomaterial

A wound-dressing biomaterial based on salmon-gelatin was fabricated and sterilized using our methods previously reported [10]. Briefly, a solution of salmon-gelatin with excipients was prepared: salmon-gelatin 0.6% *w*/*v*, chitosan 0.2% *w*/*v*, agarose 0.2% *w*/*v* and glycerol 0.1% *w*/*v*. The solution was mixed at 50 °C for 1 h and poured into a Petri dish. Then it was cooled at 4 °C, frozen at −80 °C and lyophilized. The dry composite was crosslinked by the use of EDC/NHS/MES/Ethanol (30 mM/8 mM/50 mM/90% *v*/*v*). The resultant crosslinked composite was washed with an ethanol/water solution, frozen and freeze-dried. Finally, the biomaterial was sterilized using gamma radiation at 25 kGy.

The full physical characterization of SGB is available in Enrione et al. [10]. Briefly, SGB has a Young’s modulus of ~170 Pa and stress at break of ~463 Pa. Thermal properties, determined by differential scanning calorimetry, shows a semicrystalline molecular ordering with well-defined glass transition and meting temperatures at ~46 °C and ~104 °C, respectively [10].

The microstructure of the biomaterial was examined using scanning electron microscopy (SEM, Carl Zeiss, EVOMA 10, Oberkochen, Germany). Samples were previously coated with gold (10–20 nm thickness).

### 2.3. Animal Procedures

The animal handling and surgical procedures were reviewed and approved by the Ethical Scientific Committee from the Universidad de Los Andes, Santiago, Chile on 11 December 2017 as stated in the document N^o^ CEC201753. Three female pigs (*Sus scrofa*, Yorkshire, 12 weeks, ~20 Kg) were used. After acclimatization in the animal facility for one week, the animals were anesthetized and prepared for surgery.

Animals were sedated with acepromazine-xylazine mix (2 mg/kg). Then, they were anesthetized with zoletil^®^ (tiletamine and zolazepam commercial mix) at a dose of 4 mg/kg [14,15,16]. A selected dorso-lumbar area was shaved and disinfected with povidone-iodine solution. Two cutaneous excision wounds of 3 cm in diameter were performed. Subsequently, the biomaterial was implanted over one of the wounds, leaving the other wound as a control. The latter was covered with a commercial wound-dressing product (Suresite 123, Medline, Northfield, IL, USA). All wounds were then covered with a layer of gauze, to prevent the detachment of the materials. A third wound without treatment as negative control was discarded in order to avoid infections during the study that could have affected the animal recovery. 

After surgery, the physiological evolution of the animal was followed every day for four weeks. Animal growth, physiological changes and evolution of wound healing process were evaluated in all three individuals. Photographic image assessment of each animal wound was performed once a week. At the end of the study (four weeks), the pigs were euthanized according to the approved guidelines to proceed with a histological analysis of the full-thickness skin sections [17].

### 2.4. Histological Analysis

The full-thickness biopsies of the porcine skins were fixed in Bouin aqueous for 48 h. After washing with ethanol 70%, they were cut in two halves (left and right) on the axis perpendicular to the scar, on a cutting axis parallel to the cephalo-caudal axis of the animal. Both halves for each implant were processed by routine histological technique. Histological sections obtained with microtome (5 µm) were stained with Arteta’s trichrome stain (Hematoxylin, Erythrosine-Orange G, Blue Aniline). Briefly, sections were stained in Harris haematoxylin solution for 75 s and rinsed in tap water for 10 min, followed by a quick rinse in distilled water. They were then stained with a mixture of 0.5% Erythrosin-orange G 0.5% for 30 min, and quickly rinsed in distilled water. After a 10 min bath in 0.5% phosphotungstic acid, they were quickly rinsed in distilled water and were stained in 1% Aniline Blue for 75 s [18].

## 3. Results

### 3.1. Microstructure of Salmon-Gelatin Biomaterial

**Figure 1:**
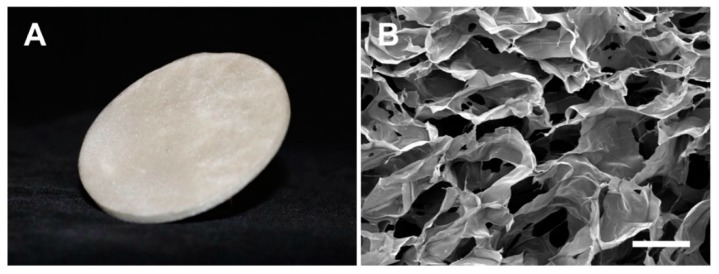
Salmon-gelatin biomaterial. (**A**), photographic image. (**B**), SEM image (Bar 200 µm).

Figure 1 shows the salmon-gelatin biomaterial (SGB). The scanning electron microscopy (SEM) shows a regular porous structure (Figure 1B), comprising an average pore diameter of ~160 μm.

### 3.2. In Vivo Assessment of Salmon-Gelatin Biomaterial

**Figure 2:**
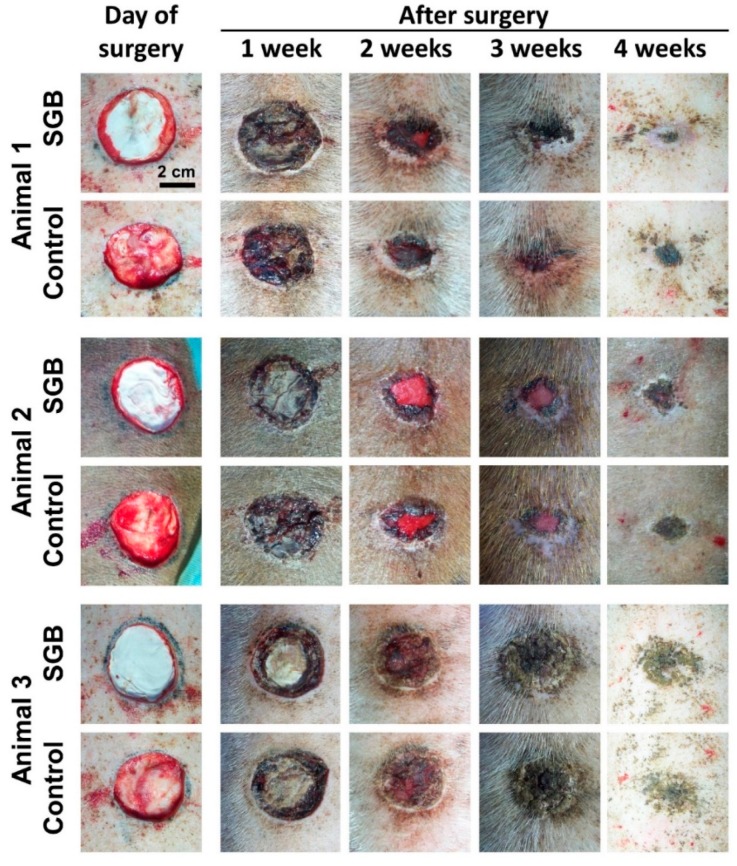
In vivo trial of wound healing process in porcine model (Bar 2 cm).

Figure 2 shows the temporal evolution of the healing process in the porcine model over four weeks. The healing process do not show apparent symptoms of inflammation or infection, which demonstrates the adequacy of animal surgery and handling, in which small controlled wounds produced during care did not show detrimental experimental signs [13].

**Figure 3:**
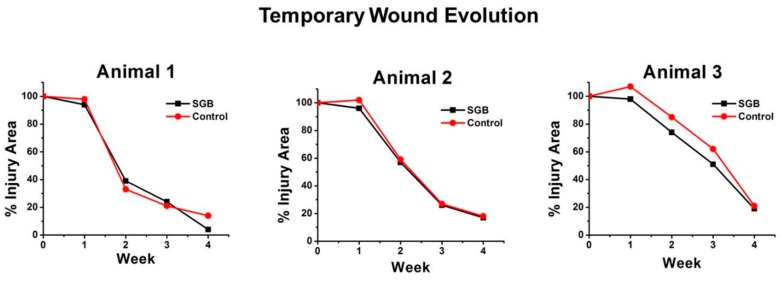
Evolution of the size of wounds (% with respect to initial area) during 4 weeks.

Figure 3 shows the temporal evolution of wound sizes for the three animals. For subjects animal 1 and animal 2, there were no relevant differences in wound size evolution between SGB and control. For animal 3, the healing process with SGB was evidently faster than the control.

### 3.3. Histological Analysis

**Figure 4:**
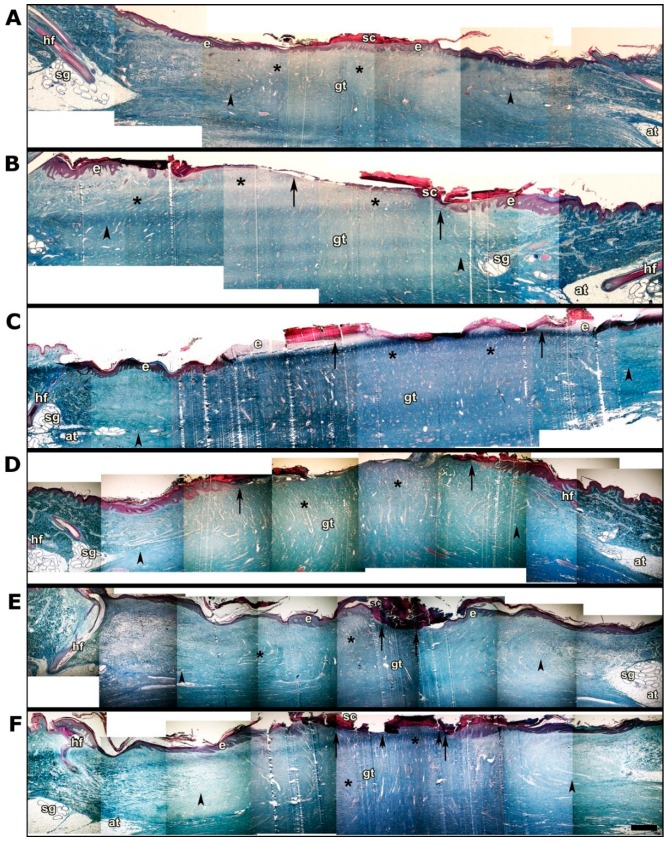
Histological study after 4 weeks of wound dressing implantation. Panoramic view showing the entire scar area. (**A**,**C**,**E**) correspond to the SGB biopsies of animals 1, 2 and 3, respectively. (**B**,**D**,**F**) correspond to the control (commercial wound dressing) biopsies of animals 1, 2 and 3, respectively. Hair follicles (hf), sweat glands (sg), granular tissue (gt) are shown. The arrowheads indicate the extent of granular tissue. The arrows indicate the extent of the healing surface. Asterisks indicate areas with high blood capillary and sinusoid content. Bar 1 mm.

Photomicrographs show histological slices of healing areas in the skin of the three pigs that were implanted (Figure 4). A–F: Panoramic view showing the entire scar area and the edges with normal skin with hair follicles (hf) and sweat glands (sg) projected towards the surface from the adipose tissue hypodermis. The arrowheads indicate the extent of the granular tissue. The arrows indicate the extent of the healing surface without epidermal lining of the biopsied skin. Asterisks indicate areas of high blood capillary and sinusoid content in the granular tissue (gt).

**Figure 5:**
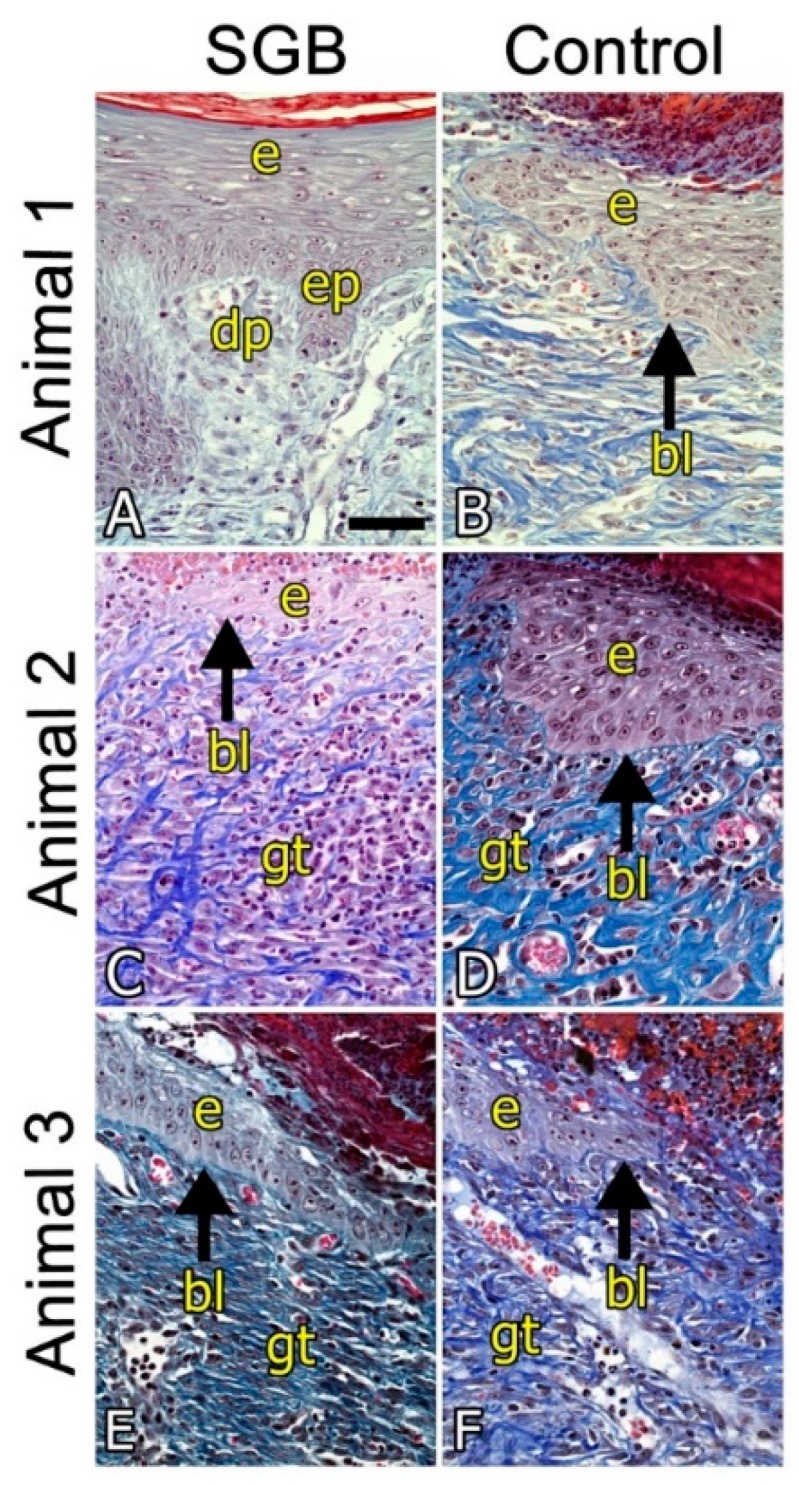
Histological study after four weeks of wound dressing implantation. Amplification of photomicrographs showed in Figure 4. Epidermal epithelial lining (e), basal lamina (bl and arrow), epidermal papillae (ep), dermal papillae (dp) and granular connective tissue (gt) are shown. Bar 50 µm.

Figure 4A,B shows photomicrographs of the SGB and the control biopsies (commercial wound dressing) of animal 1, respectively. Figure 4A shows scarring with full epidermal lining, whereas inset B shows an area without epidermal lining and a greater extension of granular tissue (11.8 mm in the control versus 10.5 mm in the GBS treated wounds). The effectiveness of tissue repair in this individual is even more evident in Figure 5A (SGM biopsy) and Figure 5B (commercial biopsy). The conformation of the epidermal lining which consists of a keratinized stratified squamous epithelium is clearly distinguished, to which underlies the basal lamina (lb), with epidermal papillae (ep) that invaginate and interdigitate with dermal papillae (dp) of loose connective tissue.

Figure 4C,D shows the photomicrographs of animal 2. Both pictures show an incomplete epidermal lining and vascularized granular tissue, although both slightly larger in the case of the SGB biopsy (17.4 mm in the SGB biopsy versus 13.9 mm in the control biopsy) that would indicate healing process in a more advanced stage. This result was also evidenced after measurement of the area without epidermal lining in the biopsies; 7.3 mm in the SGB and 8.6 mm in the control. Small differences in the conformation of the tissue structure are also more evident in the enlarged photomicrographs in Figure 5C,D.

Figure 4E,F shows the photomicrographs of animal 3. Similarly, to animal 2, the SGB biopsy shows a wound healing process profile that is slightly better than the control biopsy, with a larger region of epidermal lining. Indeed, the area of the biopsies measured without epidermal lining was 1.4 mm in SGB and 4.2 mm in the control. Also, a slightly greater extension of granular vascularized tissue was observed (14.3 mm in SGB biopsy and 14.0 mm in the control). Histological differences between SGB and control are also noticeable in the enlarged photomicrographs showed in Figure 5E,F.

## 4. Discussion

In this research, we studied the use of a wound-dressing biomaterial based on salmon-gelatin, with regular porous microstructure, on the healing process and re-epithelialization in a porcine skin model. This research confirms and validates the results obtained previously using a rabbit skin model [10], gathering additional preclinical information aiming for biosafety and effectiveness of the biomaterial required for phase I clinical trial.

In comparison to dermal wound healing process in humans, porcine skin featuring full thickness excisional wounds is by now a widely accepted model [19], mainly by the conformation, dimensions and mechanism of repair of the skin. In addition, this model allows the generation of small controlled wounds that do not activate relevant inflammatory processes, which is demonstrated in the present study [13]. 

Our results could establish that wounds treated with SGB present at least equivalent healing and epithelialization profiles with respect to control featuring a commercial product as dressing. As shown in Figure 2, variability among the studied animals was established regarding the healing process. When using farming animals, there is a probable genetic variability that results in different healing profiles, which establishes a restriction in the scope of the results. This fact has been evidenced by different authors, but it is also valuable since it can be considered a reflection of the variability of dermal physiology in humans [20,21,22]. 

Histological analysis showed results that corroborate the adequacy of using SGB for skin wound treatment. Briefly, the skin healing process develops in four critical phases; hemostasis, inflammation, proliferation, and maturation, in each one of them it is possible to observe evident histological changes, which can be studied in detail [12,23]. In our investigation, after four weeks of treatment, the histology of the skin samples showed that the analyzed tissues were in the proliferation phase. In this stage there are two characteristic processes, the formation of vascularized granulation tissue and epithelialization, the latter prior to the final stage of maturation [24,25]. 

In general, the benefits of the use of gelatin are well-known, and include improving cell adhesion and proliferation, biodegradability and non-immunogenicity [26]. However, there are substantial differences according to their origin. In this sense, the gelatin from cold water adapted fish species has a lower proportion of proline and hydroxyproline featuring particular physicochemical properties that have an impact to the gelling processes, which can impact the final mechanical characteristics of the materials conformed with these gelatins [27,28,29]. For this reason, both the formulation of nanocomposites and their crosslinking are fundamental to the success in the use of the biomaterial [8,30,31,32]. In this respect, our formulation allows cell viability to be maintained as well as reducing the possible effects on the material microstructure after sterilization by gamma radiation [33,34]. The results of this research confirm the capabilities of wound-dressing biomaterial based on salmon-gelatin for tissue engineering. 

Considering concerns for the transmission of infective-related diseases from other animal sources [26,35,36] and the interest in adding value to by-products generated by the fish industry [37,38], the data presented in this manuscript provide important background, which together with available literature, augur a positive development towards the generation of biomaterials from fish derived materials.

## 5. Conclusions

A novel biomaterial based on gelatin produced from salmon skins was evaluated in a porcine skin model as wound dressing for rapid and safe skin repair. Using a commercial product as control for comparative purposes, the present investigation demonstrates that SGB has at least the same capabilities in terms of the regenerative and biocompatibility characteristics. The latter foresees the feasibility of developing new therapeutic alternatives for skin repair based on this biomaterial produced using low cost production protocols.
